# Influence of artificial aging and ZrO2 nanoparticle-reinforced repair resin on the denture repair strength

**DOI:** 10.4317/jced.56610

**Published:** 2020-04-01

**Authors:** Mohammed M. Gad, Ahmed Rahoma, Reem Abualsaud, Ahmad M. Al-Thobity, Sultan Akhtar, Intisar A. Siddiqui, Fahad A. Al-Harbi

**Affiliations:** 1 MSc. College of Dentistry, Imam Abdulrahman Bin Faisal University, P.O. Box 1982, Dammam 31411, Saudi Arabia; 2 PhD. College of Dentistry, Imam Abdulrahman Bin Faisal University, P.O. Box 1982, Dammam 31411, Saudi Arabia; 3 DScD. College of Dentistry, Imam Abdulrahman Bin Faisal University, P.O. Box 1982, Dammam 31411, Saudi Arabia; 4 FRCD(C). College of Dentistry, Imam Abdulrahman Bin Faisal University, P.O. Box 1982, Dammam 31411, Saudi Arabia; 5 PhD. Department of Biophysics, Institute for Research and Medical Consultations (IRMC), Imam Abdulrahman Bin Faisal University, P.O. Box 1982, Dammam 31411, Saudi Arabia; 6 DScD, FACP. College of Dentistry, Imam Abdulrahman Bin Faisal University, P.O. Box 1982, Dammam 31411, Saudi Arabia

## Abstract

**Background:**

The purpose of this study was to evaluate the effect of aging process on the tensile strength (TS) of repaired acrylic denture base using ZrO2 nanoparticles (nano-ZrO2)-reinforced autopolymerized resin.

**Material and Methods:**

A total of 240 heat-polymerized acrylic resin specimens (n=10) were prepared and sectioned creating 2 mm-repair-gap. Autopolymerized acrylic resin, pure and modified with 2.5, 5, and 7.5wt% nano-ZrO2 were used for specimens repair. TS of repaired specimens were measured using the universal testing machine after water immersion at 37oC for 2, 7 and 30 days. At each time interval, half the immersed specimens underwent thermo-cycling aging process (5000 cycles at 5/55°C) before TS testing. One-way ANOVA and Tukey-Kramer multiple-comparison tests were used for data analysis at α=0.05.

**Results:**

Aging process for all groups showed significant differences in TS between unreinforced and nano-ZrO2 reinforced groups (*p*<0.05). Within immersed nano-ZrO2-reinforced specimens, 5% group immersed for 30-days showed the highest significant TS value (*p*<0.05). With regards to thermocycling, 5% group showed the highest TS values after 2-days and 30-days groups while after 7-days, significant differences were found between 2.5% group and 5% and 7.5% groups (*p*<0.05). SEM images analysis displayed the ductile fracture type for nano-ZrO2 reinforced groups.

**Conclusions:**

In summary, 5.0%-nano-ZrO2 addition to repair resin showed an improvement in tensile strength of repaired acrylic resin with different aging processes.

** Key words:**Acrylic resins, denture repair, tensile strength, thermocycling, water storage, zirconium oxide nanoparticle.

## Introduction

Poly (methyl methacrylate) (PMMA) has been used as the most common material for the fabrication of removable dentures due to their favorable characteristics, ease of handling and pleasing aesthetics. However, deficiency in mechanical properties including strength could affect the longevity of dentures (1). Intra-orally, denture base is subjected to different types of stresses leading to fatigue and eventually denture fracture (1,2). Construction of a new denture is an expensive and lengthy procedure requiring multiple clinical visits; for this reason, denture repair is recommended (2). Denture repair material should have adequate strength, dimensional stability, good color match, and the repair procedure to be easily and quickly performed (3). A major issue with denture repair is that it results in a weaker denture than the original and may re-fracture shortly. Moreover, repeated fractures are troublesome, causing economic loss and patient discomfort (2,4).

Amongst factors that affect the repair strength is the repair material type (3). The most popular repair material is auto-polymerizing acrylic resin, because it offers simple and rapid repairs. Unfortunately, it doesn’t fulfill the requirements to restore the denture original strength and avoid further fracture (5). The insufficient strength of auto-polymerizing acrylic resin compared to that of heat-polymerizing acrylic resin is the main reason for this unfavorable phenomenon (3). Therefore, numerous methods to enhance the strength of the repaired parts have been reported including repair material reinforcement (3,6). The inclusion of different reinforcing materials into repair acrylic has been tried and recognized in the literature. Reinforcements included stainless steel wires, alloy mesh, and fibers (6).

Nowadays, nanotechnology invaded this field improving the properties of denture repair. ZrO2 nanoparticle (nano-ZrO2) is a metal oxide that is biocompatible, has good mechanical strength and favorable surface properties making it a suiTable acrylic reinforcing material, including repair resin of denture bases (7,8). Recently, Gad *et al.*, reported that the incorporation of nano-ZrO2 into PMMA denture base significantly improved the repair strength and this enhancement in strength was dependent on the application and manipulation (9,10). Additionally, good adhesion and homogenous distribution of nanofillers within the resin matrix effectively improve the nanocomposite properties. Additionally, the use of silane coupling agent to treat the nano-ZrO2 surface may reduce the nano-filler agglomeration and improve the distribution within polymer matrix (10).

Denture repair success depends on the durability of materials used, which could show signs of decline strength reducing the longevity of the prosthesis. During clinical use, dentures are either immersed in saliva or stored in water or denture cleansers (11). Relatively, PMMA absorbs small amounts of water when stored in moist environment. However, this small amount has the ability to significantly affect the mechanical and dimensional properties of the polymer (12). According to Takahashi *et al.*, (13) the reaction of the denture base to absorbed water is unlike that of repair material due to differences in chemistry and water sorption ability. Accordingly, the strength of water-sorbed resin is dependent on the intrinsic mechanical properties of the material and the amount of water imbibed (13). Dentures in use are exposed to a range of temperatures within a wet environment, (14) and therefore, it is imperative to verify whether these changes in temperature could affect the mechanical properties of dentures. Recommendations to include thermal cycling as part of the testing protocol for dental polymers were made. Thermal cycling is an in-vitro procedure to simulate the conditions of oral cavity, in which tested materials are exposed to alternating extreme temperatures using thermally controlled water baths. Thus, the influence of thermal cycling on mechanical properties of repaired denture bases must be studied to determine its impact on clinical performance (8).

In view of the deficiencies of auto-polymerized PMMA that include poor mechanical properties and vulnerability to fracture, nano-ZrO2 was added to overcome these deficits. However, there is little information on the tensile strength (TS) of repair resin reinforced with nano-ZrO2. Additionally and to the authors’ knowledge, the effects of water immersion and thermal stressing have not been previously reported. Therefore, this in-vitro study aimed to evaluate the effects of water immersion and thermal cycling on repaired denture base using repair material reinforced with different concentrations of nano-ZrO2. The null hypothesis of this study was that water immersion and thermal cycling had no effects on TS of repaired denture base resin.

## Material and Methods

-Specimen preparation 

Heat-polymerized acrylic resin was used to fabricate 240 dumbbell-shaped specimens according to Specification no. 12 of the American Dental Association for denture base polymers (15). A split press metal mold was prepared with internal dimensions of (32 × 6 × 2.5 ± 0.03 mm). Acrylic resin specimens were fabricated using the conventional method for denture construction. Molten wax was used to wax-up the mold. Then, wax specimens were invested in dental stone using metal flasks. After stone setting, the flasks were placed into wax elimination machine for 10 min to melt the wax and create empty mold spaces. Separating medium was painted on stone surfaces while still warm. Following the manufacturer recommendations, heat-polymerized acrylic resin (Major base 20, Major Prodotti Dentari, SPA, Italy) was mixed and packed into mold spaces at the dough stage. The two parts of the flask were closed under pressure using a hydraulic bench press for 5 minutes then flask was allowed to bench set for 30 minutes before polymerization. Acrylic resin was heat-polymerized using water bath by heating to 74°C for 90 min, then 100°C for 30 min. After processing, the flask was allowed to cool slowly to room temperature then opened. Acrylic specimens were retrieved, finished and polished. Excess resin was removed using progressively finer grits of silicon carbide papers (Grits 120 to 500) with copious amount of water followed by polishing using rag wheel and pumice on a dental lathe. A digital caliber was used to evaluate the proper dimensions of all dumbbell-shaped specimens and approved specimens were kept in distilled water at 37oC for 48±2 hours.

-Preparing specimens for repair 

To standardize the specimens’ dimensions, a positional jig was used to prepare a 2-mm-repair gap. Each specimen was secured into the jig and marked on both ends for ease of reassembling. A line perpendicular to the long axis of the specimen was drawn at the center of each specimen, followed by two lines at 1-mm-distance from the center on each side. Lines were extended on the surface of the mold as a standardized guide for the rest of specimens. Cuts were made at these lines using a low speed diamond disc (DeguDent, GmbH, REF 59903107, Dentsply, Germany) and copious amount of water. This process simulates roughening of the denture base surface with laboratory burs. Specimens were randomly divided into 3 groups according to aging process. Each group was divided into subgroups according to nano-ZrO2 concentration within repair resin (Table 1).

**Table 1:**
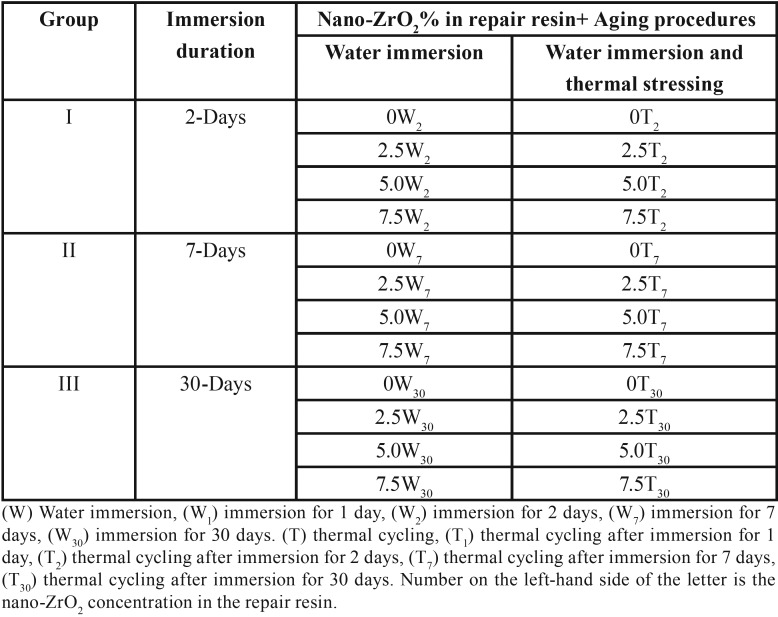
Specimen grouping and coding according to nano-ZrO2 concentration, duration of water immersion, and thermal cycling.

-Silanization of nano-ZrO2 particles and PMMA/ZrO2 nanocomposite preparation

The nano-ZrO2 powder (99.9%purity, Shanghai Richem International Co., Ltd. China) used in this study had an average size of 40 nm which was confirmed using scanning electron microscopy (SEM) and transmission electron microscopy (TEM). Creating reactive groups on the surface of nano-particles using the silane coupling agent [3-(trimethoxysilyl) propyl methacrylate (TMSPM) (Shanghai Richem International Co., Ltd. China)] could promote filler adhesion to the resin matrix (9,10). To achieve this, 0.3 g of TMSPM was dissolved in 100 ml of acetone followed by the addition of thirty grams of ZrO2 nanoparticles. The mixture was stirred using a magnetic stirrer (Cimarec Digital Stirring Hotplates, SP131320-33Q, Thermo Scientific, China) for 1 hour. Next, the solvent was removed under vacuum using a rotary evaporator at 60°C and 150 rpm for 30 min. The dry powder was then heated to 120°C for 2 h and cooled to room temperature to produce the surface-treated nano-ZrO2 (16). The silanized nano-ZrO2 was weighed using an electronic balance and added in concentrations of 2.5wt%, 5wt%, and 7.5wt% of auto-polymerized acrylic powder (9,10) (Major repair, Major Prodotti Dentari, SPA, Italy). The pre-weighed silanized nano-ZrO2 was thoroughly mixed with acrylic powder for 30 min to achieve a mixture with uniform color and homogenous distribution of nanofiller (17).

-Repair procedure

Repair procedure was performed in a similar manner to that used to repair denture bases of complete and partial dentures. Specimen surfaces were treated with monomer for 180 seconds. Pairs of each specimen were placed into the mold and fixed keeping 2 mm-repair-gap. According to manufacturer’s instruction, repair acrylic was mixed in a ratio of 10 g powder to 7 ml liquid. The free-flowing mix was poured into the repair gap insuring a slight overfill to compensate for polymerization shrinkage, finishing, and polishing. Polymerization was completed in a pressure pot at 45oC for 15 min and 2.2 bar of pressure. After polymerization, specimens were finished to restore the original dimensions using 600 grit silicon carbide papers. Polished specimens were then kept in distilled water in four main containers according to subgroups (nano-ZrO2 concentration in the repair resin). Control group (pure repair resin per group and time interval - 0W) was tested after two days of water immersion. After two days (W2), 40 specimens (10 per nano-ZrO2 concentration, 0W2, 2.5W2, 5.0W2, 7.5W2) were randomly selected and tested. Forty more specimens (0W7, 2.5W7, 5.0W7, 7.5W7) were randomly selected and tested after 7 days and the last 40 specimens (0W30, 2.5W30, 5.0W30, 7.5W30) were tested after 30 days. The remaining half of specimens (130 specimens- n=10) were subjected to thermal stress where they were placed in a thermal cycling machine (Thermocycler THE-1100 - SD Mechatronik GmbH, Feldkirchen-Westerham, Germany) and stressed for 5,000 cycles at 5°C and 55°C with 30-second dwell time after each immersion interval (2, 7, 30 days) (18).

-Tensile strength test

Repaired specimens were aligned vertically and gripped at both ends on a universal testing machine (Instron 8871; Instron Co., Norwood, MA, USA). Specimens were tension loaded using a 5 kN load cell at a crosshead speed of 5 mm/min until failure. The formula: TS=F/A was used to calculate TS in (MPa) where, TS= tensile strength (N/mm2), F= load at failure (N), A= cross sectional area at fracture site (mm2).

-Scanning Electron Microscopy (SEM)

Scanning electron microscope (SEM) (FEI, INSPECT S50, Czech Republic) was used to inspect the surface of acrylic specimens and the cross-sections at fracture. The specimens were gold coated using a sputter coating machine (Quorum, Q150R ES, UK). Coated specimens were scanned at 20 kV with a working distance of ~ 10 mm and 3.5 spot size capturing images at various magnifications to analyze important features of specimens’ failure mode.

-TEM results of ZrO2 nanoparticles 

TEM was used to estimate the size of nano-ZrO2 particles. The diameter of more than 80 particles was measured by extracting the intensity profiles and the average size was found to be ~ 40 ± 2 nm.

-Statistical analysis 

SPSS-20.0 (IBM, Armonk, NY) was used to complete statistical data analysis. Results of the TS test were tabulated and represented in means and standard deviations (SD). Comparisons of TS values between groups (after different durations of water immersion) and within different groups (nano-ZrO2) relative to that of control were done. One-way ANOVA and Tukey-Kramer multiple-comparisons tests were used. Results with *p*-value ≤ 0.05 were considered statistically significant.

## Results

Means and standard deviations for TS values after water immersion were summarized in Table 2. Among unreinforced group (0W2, 0W7, 0W30) and after different water immersion periods, results showed no significant difference (*p*>0.05) where 0W2 had the lowest TS value (39.88 ± 2.72 MPa). Within reinforced groups and after 2-day-immersion, significant differences were found between (2.5W2/5.0W2), and (2.5W2/7.5W2) (*P*<0.05) with no significant difference between (5.0W2/7.5W2) (*P*>0.05). The results at the end of 7-day-immersion revealed non-significant differences between 2.5W7, 5.0W7 and 7.5W7 (*P*>0.05). After 30-day-immersion, significant differences were found between (2.5W30/5.0W30) and (5.0W30/7.5W30), with no difference between (2.5W30/7.5W30) (*P*>0.05). Among all reinforced groups, 5.0W30 showed the highest TS value (70.82 ± 2.74 MPa) while the lowest TS value was reported with 2.5W2 (57.54 ± 3.25 MPa).

**Table 2:**
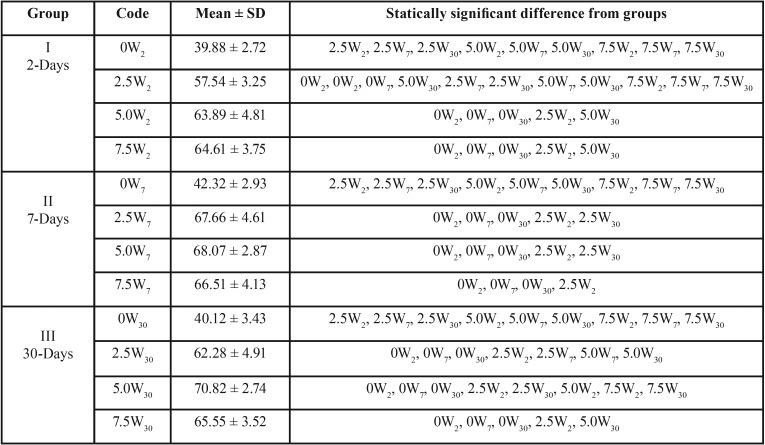
Tukey-Kramer Multiple-Comparison test for tensile strength (MPa) of denture base resin showing mean ± SD, and groups with significant differences after water immersion.

The means and standard deviations of the TS values for specimens undergoing thermal cycling were summarized in Table 3. Between unreinforced groups (0T2, 0T7, 0T30), no significant difference in TS values with thermal stress were observed between 0T2, 0T7 and 0T30 (*P*>0.05) where group 0T30, had the lowest TS value (28.45 ± 3.97 MPa). Thermal stress after 2-day-immersion (T2) showed significant differences between (2.5T2/5.0T2) and (2.5T2/7.5T2) (*P*<0.05) with no significant difference between (5.0T2/7.5T2) (*P*>0.05). Thermal stress after 7-day-immersion (T7) resulted in significant differences of TS values between (2.5T7/5.0T7) and (2.5T7/7.5T7) (*P*<0.05) with no significant difference between (5.0T7/7.5T7) (*P*>0.05). At 30-day-immersion (T30), thermal stress resulted in significant differences between (2.5T30/7.5T30) and (5.0T30/7.5T30) (*P*<0.05) with no detected difference between (2.5T30/5.0T30) (*P*>0.05). Between all reinforced, thermo-cycled groups, 5.0T30 exhibited the highest TS value (61.44 ± 4.18 MPa) while 2.5T2 had the lowest TS value (49.85 ± 4.98 MPa).

**Table 3:**
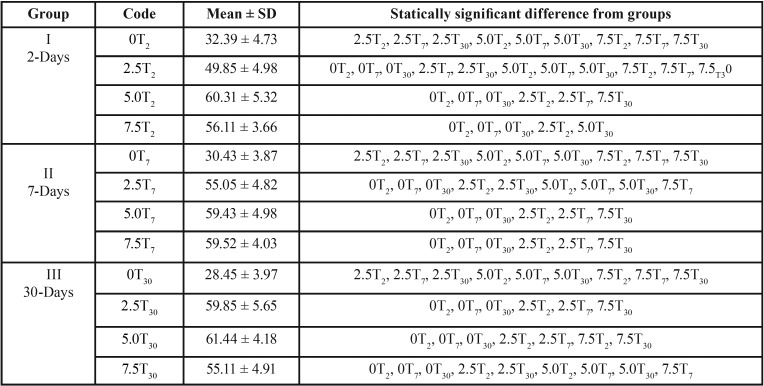
Tukey-Kramer Multiple-Comparison test for tensile strength (MPa) of denture base resin showing mean ± SD, and groups with significant differences after thermal cycling.

**Figure 1:**
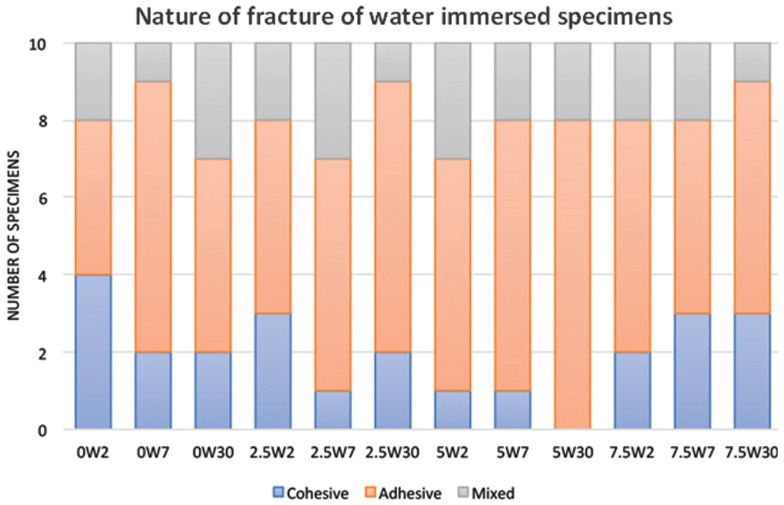
Analysis of nature of fracture after tensile strength testing of specimens undergoing water immersion treatment.

**Figure 2:**
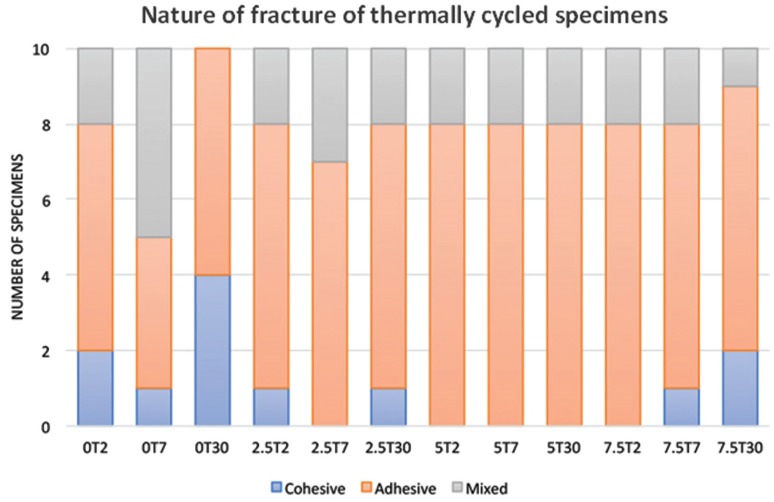
Analysis of nature of fracture after tensile strength testing of thermo-cycled specimens.

Figures 1 and 2 showed the variations in nature of specimens fracture. With water immersion at different intervals, there were variations in nature of fracture. Most common fracture type was adhesive followed by cohesive then mixed. With nano-ZrO2 reinforcement, the most frequent type of failure was adhesive even at higher TS values. With thermal cycling aging, adhesive failure was the most common type of fracture for all tested groups.

**Figure 3:**
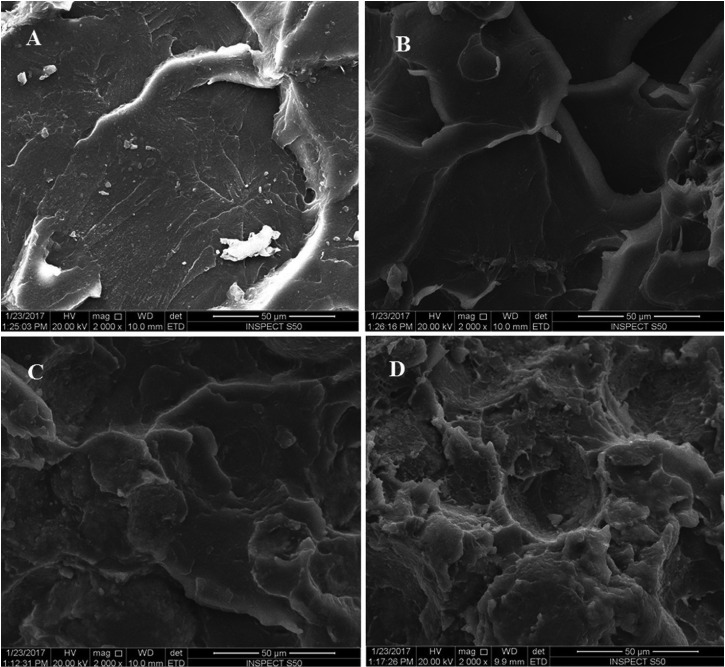
Representative SEM Images of fractured surfaces. (A) Control; (B) 2.5% Nano-ZrO2; (C) 5% Nano-ZrO2; (D) 7.5% Nano-ZrO2.

The morphology of the fractured surfaces of different samples was observed by SEM. Figure 3 shows the representative images of PMMA/nano-ZrO2 composites and the surface characteristics of fractured specimens. In control group (I), brittle fracture with smooth fracture surfaces and few irregular trabeculae were observed (Fig. 3A). At 2.5% nano-ZrO2, the fracture surface was relatively irregular with scattered lamella in addition to the presence of nano-ZrO2 evenly dispersed in the matrix (Fig. 3B). At 5% nano-ZrO2, nanoparticles were dispersed evenly in the matrix with small clusters and pits starting to scarcely appear (Fig. 3C). The fracture surface had more irregular lamellae and steps representing ductile fracture. At 7.5% nano-ZrO2, loosely attached clusters were formed and large voids and pits started to appear (Fig. 3D). The filler particles were distributed well within the resin matrix and the fracture surface had more irregular lamellae and steps representing ductile fracture. Nano-ZrO2 clusters at some instances were seen on one fracture side leaving voids on the other side.

## Discussion

Removable prostheses are subjected to variety of intraoral conditions that may cause structural and dimensional changes. The humid intraoral environment in addition to fluctuating temperatures may facilitate water sorption into the resin (14). Therefore, evaluation of long-term water immersion and thermal stressing on mechanical properties of repaired denture base resin is of utmost benefit; however, this has not been investigated until now especially with repair denture bases modified with nano-ZrO2. After evaluation of the results, the null hypothesis was rejected where water immersion and thermal cycling affected the TS of denture base repaired with nano-ZrO2 modified repair resin.

The water immersion resulted in a slight increase in TS after 2- and 7-days, followed by a decrease after 30-days. It is worth noting that those effects were insignificant. The humid intraoral condition is capable of enhancing water sorption into the resin. It was reported in the literature that equilibrium after water sorption takes 24 h. However, given the results of the present study; 0W2 group appeared to reach saturation level after 48 hours of immersion. When the denture base is submerged in water, unreacted monomer, plasticizers and other soluble components may leach out (19). The resulting micro-voids are filled with the absorbed water molecules. Both processes; effusion of soluble constituents and infusion of water, are time-dependent, and the amount of sorbed water changes until equilibrium is achieved (12). This increase in TS may also be explained by the continued polymerization of un-reacted monomer after the initial polymerization which increases the degree of conversion of resin. However, water sorption may have overcome the effects of continued polymerization at the initial stages of immersion (12). In-between the end of 7-days and beginning of next 21-days, it can be hypothesized that the specimens have reached the level of water saturation, and that the increase in TS is due to the surge in reaction of double bonds of radicals (19).

With prolonged immersion time (30-days), TS deceased and this may be attributed to water sorption, plasticizers, and unreacted monomers negatively affecting the mechanical properties of denture base through facilitation of resin chain movements. Hence, the strength of the denture base after water immersion is dependent on the amount of these molecules (12,20). The imbibed water has the ability to affect both mechanical and dimensional properties of the resin. The introduction of water within polymerized resin mass results in two important phenomena. First, it acts as a plasticizer, and second, it causes expansion of the resin mass (13,20). The plasticizing effect of water allows the polymer chains to slide over one another with ease; thus, reducing mechanical properties of denture base and repair resin (11,12,21).

Denture base and repair resin are normally exposed to varying temperatures in the wet oral environment (14,21). One of the desirable properties of repaired denture bases is good level of fracture resistance. Hence, it is imperative to determine the effect of fluctuating temperatures on the mechanical properties of these resins due its paramount clinical significance. To simulate this unique environment, thermal cycling is recommended to be part of the dental polymer testing protocol (21). Therefore, specimens in this study were thermally cycled (5,000 cycles at 5°C/55°C) in water baths prior to tensile testing. According to the results of this study, the hypothesis that TS of acrylic resins evaluated would not be affected by thermal cycling was partially accepted. Thermal cycling procedure allows for not only evaluating the effect of fluctuating temperatures but in addition, it allows for water immersion (8). The water absorption combined with temperature changes may lead to denture base degradation. The water molecules infiltrate the PMMA filling the inter-polymeric chain spaces and forcing the chains apart, which in turn affects the mechanical properties of the denture resin (8).

During thermal stressing, high temperatures may accelerate the ingress of water, increasing its plasticization effect and reducing resin mechanical properties. Results of this study showed a significant decrease in the TS with thermal cycling combined with intervals of water immersion. There was an inverse relation between TS and thermal stresses after different immersion time. The TS decreased after water immersion combined with thermal stress than water immersion alone. This decrease may be attributed to water intake. Water uptake is significantly increased at high temperatures, which leads to supersaturation of the acrylic surface during cooling of the specimen (22). A previous study investigated the flexural and impact strengths of acrylic denture base resin after 5,000 cycles of thermal stressing and found a significant decrease in both strengths (21). The absorption of water into the resin is affected by the polarity of PMMA and the ability of water molecules to diffuse into interstitial spaces around polymer chains (23). Thermal cycling may increase the amount of sorbed water by increasing the volume of interstitial spaces or increasing the rate at which water molecules diffuse into the denture base (24).

Nano-ZrO2 addition significantly increases the TS. The homogenous distribution of nano-ZrO2 utilized in this study permitted them to fill spaces between polymer chains, thereby limiting the movement of macromolecular chains and improving the strength and rigidity of the resin. This mechanism ultimately improved the TS (25). Additionally, this positive effect of nano-ZrO2 may be attributed to a process called transformation toughening where nano-ZrO2 changes from the tetragonal phase to monoclinic phase after stressing. In this transformation, the crystals expand in size and place the crack under a state of compression arresting its propagation (25). Previously, Gad *et al.* studied the effects of nano-ZrO2 addition into repair material and found a significant increase in repair strength (9,10).

In this study, the TS determined 30-days after immersion in water and thermal stressing indicates the effectiveness of nano-ZrO2 reinforcement. Nano-ZrO2 reinforced repair material did not show significant deterioration in TS. However, even with this increase in TS compared to control group, inadequate bond strength was detected and failure through the repair site was common. Conversely, a large number of specimens in the nano-ZrO2 reinforced groups fractured at the repair interface exhibiting adhesive failure resulting from weak bond strength between denture base and repair resin (1). To increase repair bond strength, modifications such as repair surface treatment, surface design, in combination with repair material reinforcement could be a promising method for adequate repair strength. Moreover, additional investigations are necessary to determine the effects of aging on performance of repaired denture bases over longer periods of time (3,10).

Although previous studies (8,13,18,21,22) investigated different storage periods than those used in this study, the expected duration of service of a repaired denture was not replicated. Another aspect to be taken in consideration is that repaired dentures in actual clinical conditions are exposed to a multitude of simultaneous thermal and mechanical stresses. These factors may impact the TS of the denture base and repair resin. Additionally, it should be noted that *in-vivo* conditions (simultaneous thermal and mechanical stressing) differ from the in-vitro setting where specimens are exposed to each condition separately, and therefore findings should be carefully interpreted. These were the limitations of the present study and the authors think they should be considered in future investigations.

## Conclusions

Considering the limitations of the study, the following conclusions were drawn:

For unreinforced subgroups, water immersion and thermal cycling did not affect the TS of repaired denture resin.

Adding nano-ZrO2 significantly increased the TS of repaired denture resin and the highest TS value was seen with 5% and 7.5% nano-ZrO2.

Water immersion increased the tensile strength of 2.5% and 5% nano-ZrO2 groups up to 7–days immersion without noticeable effect on 7.5% nano-ZrO2 group.

Thermal cycling had different effects on different concentrations of nano-ZrO2, where TS increased with 2.5%, stayed the same with 5% and decreased with 7.5% after long immersion periods.
